# The value of multimodal ultrasonography in evaluating therapeutic response of cervical tuberculous lymphadenitis to anti-tuberculosis drugs

**DOI:** 10.3389/fmed.2023.1177045

**Published:** 2023-07-18

**Authors:** Tianzhuo Yu, Lin Zhang, Jianping Xu, Jun Meng, Xiulei Yu, Ying Zhang

**Affiliations:** Department of Ultrasonography, Affiliated Hangzhou Chest Hospital, Zhejiang University School of Medicine, Zhejiang Integrated Traditional and Western Medicine Hospital, Hangzhou Red Cross Hospital, Hangzhou, China

**Keywords:** multimodal ultrasound, tuberculous lymphadenitis, contrast-enhanced ultrasound, anti-tuberculosis treatment, cevical

## Abstract

**Purpose:**

In order to research the value of multimodal ultrasonography in evaluating therapeutic response of cervical tuberculous lymphadenitis to anti-tuberculosis drugs.

**Materials and methods:**

Sixty-one patients with cervical tuberculous lymphadenitis were enrolled in this study. Ultrasound examination was performed before systemic standard anti-tuberculosis treatment and within 1–2 months after treatment, and the patients were divided into effective group and ineffective group according to the follow-up at the sixth month. The multimodal ultrasound signs of the two groups were compared and analyzed.

**Results:**

In the effective group, there were significant differences in the maximum diameter of lymph nodes, the echo of the surrounding tissue and the enlargement of the contrast area before and after treatment (*p* < 0.05). At 1–2 months after treatment, there were significant differences in the maximum diameter, pus changes, CDFI, elasticity scores, echo of surrounding tissues, changes in enlarged and non-enhanced areas after contrast enhancement between the effective group and the ineffective group (*p* < 0.05).

**Conclusion:**

The multimodal ultrasound signs of the appearance of internal pus or non-enhancement area enlargement, enhanced echo of the surrounding tissue and enlargement after CEUS are related to poor prognosis, and may be used to evaluate the response of anti-tuberculosis chemotherapy when the size change of lymph node is not obvious in individual treatment.

## Introduction

Tuberculous lymphadenitis (TBL) is the most common form of extrapulmonary tuberculosis, with 60–90% of the disease occurring in the neck region (1, 2). In recent years, insufficient attention has been paid to TBL. *Mycobacterium tuberculosis* (MTB) may exist in lymph nodes for a long time, leading to latent tuberculosis (3, 4). At present, anti-tuberculosis drugs are the first choice for the treatment of TBL, and interventional or surgical treatment may be required when abscesses or sinuses are formed (5). Effective treatment of TBL is particularly important in the prevention and control of tuberculosis. Timely evaluation of the response of TBL to drugs will help to adjust the treatment plan to ensure effectiveness.

Therefore, the aim of this study was to compare and analyze the multimodal ultrasound images of cervical tuberculous lymphadenitis (CTL) after treatment, in order to provide non-invasive imaging evidence for early evaluation of the treatment response.

## Materials and methods

### Patients

A total of 72 patients with CTL were treated by systemic standard anti-tuberculosis treatment at our hospital between January 2020 and June 2022. After excluding three patients with allergy to first-line anti-tuberculosis drugs and eight patients who were lost to follow-up, 61 patients were enrolled in this study, aged 18–74 years (median age 30 years). Among them, 37 were females, while 24 were males. All cases were clinically confirmed by laboratory examination or pathology. All patients gave informed consent. The study protocol was approved by the Institutional Ethics Committee of our hospital, and was in accordance with the Helsinki Declaration.

Inclusion criteria were as follows: (1) complete imaging data, including conventional US and CEUS examinations; (2) age above 18 years and voluntary; (3) no history of anti-tuberculosis chemotherapy.

Exclusion criteria were as follows: (1) Recurrent tuberculous lymphadenitis; (2) HIV-infected patients; (3) allergic to ultrasound contrast agent or first-line anti-tuberculosis drugs.

### US and CEUS examinations

The Philips iU-Elite ultrasound system (Washington, USA), with L12–5 linear array transducer (Frequency range, 5–12 MHz) and L9–3 linear array transducer (Frequency range, 3–9 MHz), and Mindray Resona 7S (Shenzhen, China), with L14–5WU linear array transducer (Frequency range, 5–14 MHz) and L9–3U linear array transducer (Frequency range,3–9 MHz), were used for the whole procedure. The ultrasonic contrast agent in contrast-enhanced ultrasonography (CEUS) is SonoVue (Milan, Italy, Bracco SpA), which was diluted with 5 mL of normal saline (NS) and shaken well before use, with low mechanical index (0.06) pulse reverse harmonic imaging system in the examination. The multimodal ultrasound images of the lymph nodes such as size, internal echo, cystic necrosis, calcification, surrounding soft-tissue echo, color-flow Doppler Imaging (CDFI), which was mainly divided into avascular, peripheral, hilar, and mixe (6), elastographyic score and CEUS patterns were recorded and stored. These data of the largest lymph node were analyzed and classified by the same two senior radiologists with more than 10 years of experience.

### Methods

LN core needle biopsy was performed in all cases, and the specimens obtained subjected to histopathological examination (HPE), acid-fast bacillus (AFB) staining and laboratory examination including Gene-Xpert, MTB culture and next-generation sequencing (NGS) sometimes, any of which was positive and diagnosis confirmed. We obtained the diagnosis results from the electronic medical record system.

All cases received standardized anti-TB drug therapy (i.e., 3 or 4 first-line TB drugs): 0.3 g isoniazid once daily, 0.45 g rifampicin once daily, 0.5 g pyrazinamide three times/day, with or without 0.75 g ethambutol once daily. Multimodal ultrasound examination was performed before treatment and within 1–2 months after treatment, and the prognosis of patients was followed-up after six months of treatment.

Treatment outcomes at the end of six months were as follows: (1) Cure: complete remission of all symptoms with disappearance of lymph nodes or reduction of the largest lymph node by less than 1 cm; (2) Improvement: symptom relief, reduction in lymph node size and number; (3) Stable: symptoms subsided and the maximum residual lymph node size was ±1 cm from baseline; (4) Deterioration: appearance of new symptoms, appearance and persistence of new lymph nodes, and/or increase in the size of the largest lymph node by 2 cm from baseline (7). The first to third outcomes were defined as the effective group, while the fourth outcome and the cases transferred to surgery were defined as the ineffective group.

The multimodal ultrasound signs of the two groups before treatment and 1–2 months after treatment were compared and analyzed, including the maximum diameter, the change of pus (the long and short diameters of the maximum section), the presence and size of strong echo, CDFI, elasticity score, echo of surrounding soft-tissue and CEUS. The evaluation indicators of CEUS included enlargement after contrast, peripheral rim-like enhancement, and the changes of non-enhancement area (the long and short diameters of the maximum section). If more than one necrotic lesion was found in one lymph node, each lesion was compared separately. The size of strong echo was divided into punctate and cluster-like, with a boundary of 2 mm. Elastographic scoring system was determined on the distribution and percentage of blue area. Score 1, blue areas <10%; Score 2, blue areas <45%; Score 3, blue areas 45–90%; Score 4, >90% blue areas (8).

### Statistical analysis

The data were analyzed by SPSS 23.0 statistical software (United States, IBM). Measurement data were expressed as mean ± SD or median, and were compared by t-tests. Count data were expressed as examples (%), the two groups were compared using Chi-square test and Fisher’s exact test. Differences were considered statistically significant at *p* < 0.05.

### Results

There were 46 cases in the effective group and 15 cases in the ineffective group. The demographic characteristics of the patients are presented in Table 1.

**Table 1:** The demographic characteristics of the patients.

	Effective (*n* = 46)	Ineffective (*n* = 15)
Age (year, mean ± SD)	33.5 ± 11.9	30.9 ± 11.5
Gender (*n*)		
Male	20	4
Female	26	11
Onset time^#^ (day, mean ± SD)	11.6 ± 6.4	12.2 ± 8.8
Other symptoms (*n*)		
Fever	11	3
Night sweat	9	2
Weigh loss	2	1
Pain	7	2
Type of medicine*		
Without ethambutol (*n*)	3	1
Duration of treatment (day, mean ± SD)	35.4 ± 4.7	35.4 ± 5.8

^#^It refers to the amount of time in the electronic medical record system that patients complain of discomfort.*All cases received standardized anti-TB drug therapy, type of medicine include isoniazid, rifampicin, pyrazinamide, with or without ethambutol.

There were no significant differences in the maximum diameter of lymph nodes, presence or absence of pus, calcification, CDFI, elasticity score, echo of surrounding tissue and CEUS between the effective group and the ineffective group before treatment (*p* > 0.05). The data are presented in Table 2.

**Table 2:** Comparison of multimodality sonographic findings of the effective group and ineffective group before treatment.

	Effective (*n* = 46)	Ineffective (*n* = 15)	*p*
Maximum diameter (cm)	4.3 ± 0.6	4.2 ± 0.8	0.359
Pus			0.858
Absence	7	2	
Presence	39	13	
Strong echo			0.561
Absence	30	12	
Punctate	5	1	
Cluster-like	11	2	
CDFI			0.911
Hilar	4	1	
Peripheral	24	9	
Mixed	16	4	
Avascular	2	1	
Elastographyic score			0.856
1	3	1	
2	15	4	
3	25	8	
4	3	2	
Echo of surrounding soft-tissue			0.833
Enhanced	17	6	
Abscess formation	0	0	
Sinus	0	0	
Normal	29	9	
CEUS			
Enlargement	19	7	0.715
Rim-like enhancement	31	11	0.912
Non-enhancement area			0.804
Absence	4	1	
Presence	42	14	

In the effective group, there were significant differences in the maximum diameter of lymph nodes, the echo of the surrounding tissue and the enlargement of the contrast area before and after treatment (*p* < 0.05), which are presented in Table 3.

**Table 3:** Comparison of multimodality sonographic findings of the effective group before and after treatment.

	Before	After	*p*
Maximum diameter (cm)	4.3 ± 0.6	2.7 ± 0.7	0.000
Pus			0.129
Absence	7	13	
Presence	39	33	
Strong echo			0.161
Absence	30	21	
Punctate	5	9	
Cluster-like	11	16	
CDFI			0.251
Hilar	4	2	
Peripheral	24	17	
Mixed	16	22	
Avascular	2	5	
Elastographyic score			0.333
1	3	2	
2	15	19	
3	25	18	
4	3	7	
Echo of surrounding soft-tissue			0.017
Enhanced	17	9	
Abscess formation	0	0	
Sinus	0	0	
Normal	29	37	
CEUS			
Enlargement	19	10	0.043
Rim-like enhancement	31	26	0.283
Non-enhancement area			0.738
Absence	4	6	
Presence	42	40	

**Figure 1:**
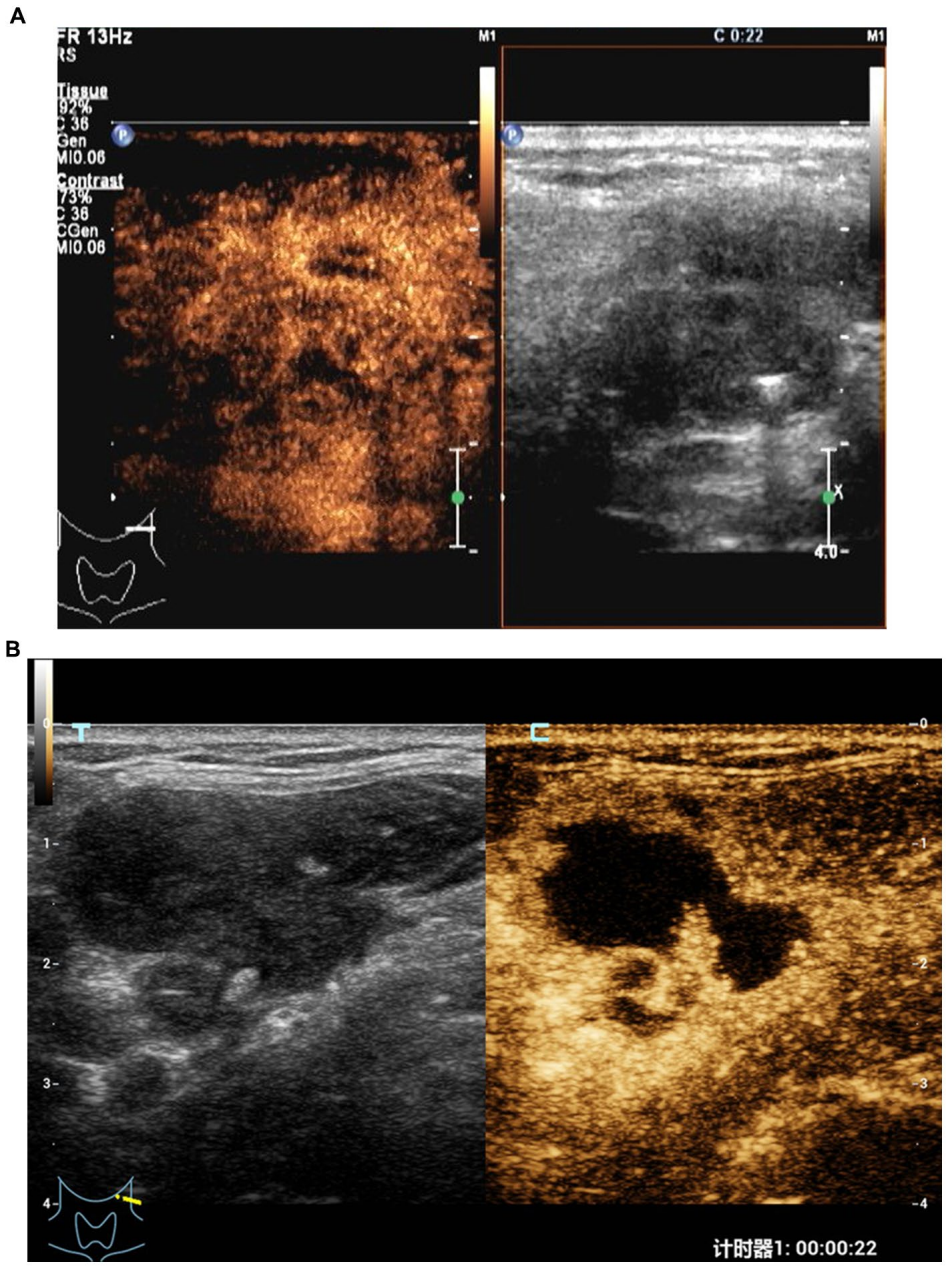
CEUS in an 33-year old woman with CTL in the ineffective group. **(A)** Before treatment, CEUS showed a small area of necrosis in the lymph nodes. **(B)** After treatment, the necrotic area was enlarged.

**Figure 2:**
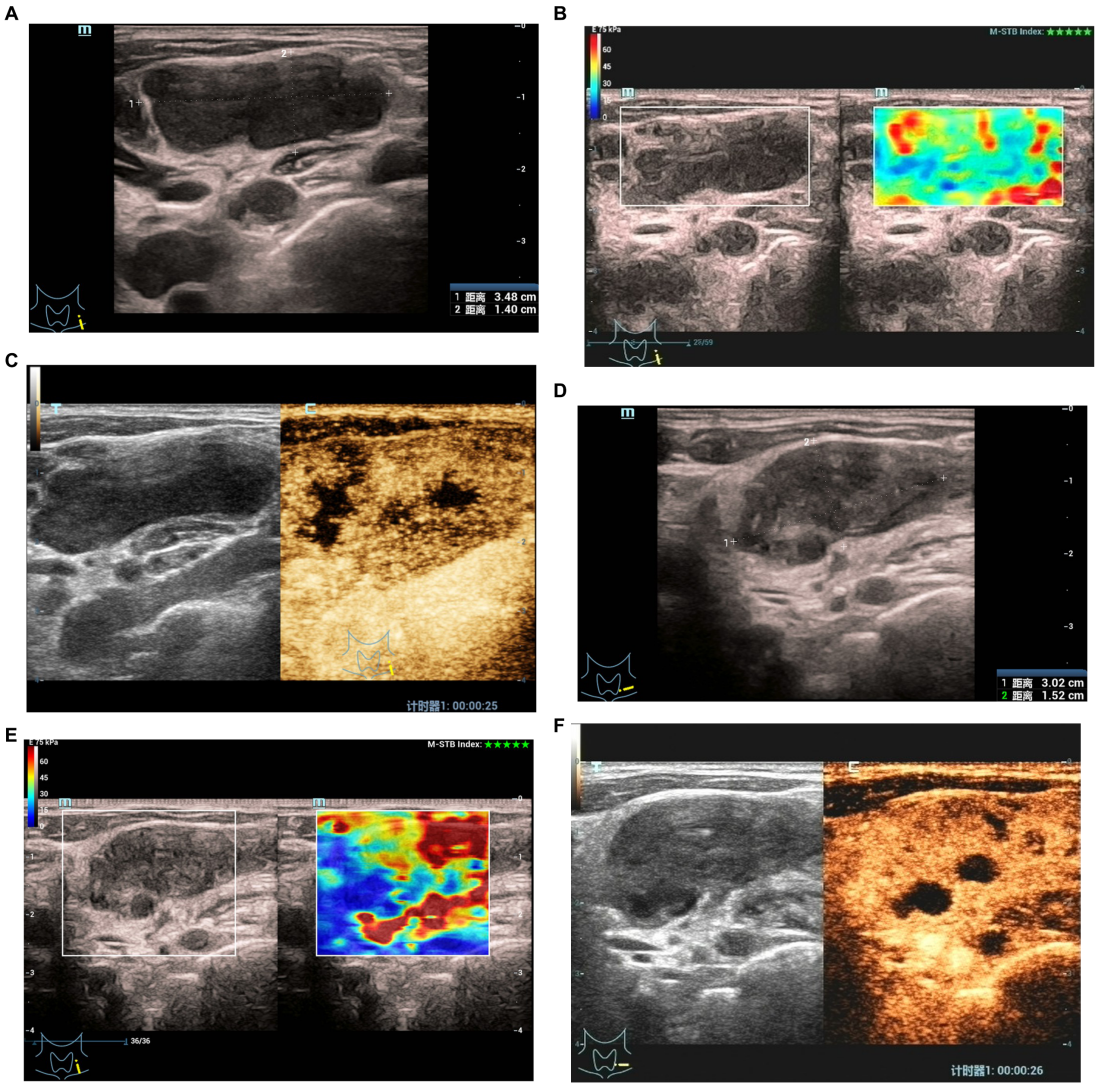
Elastographyic score and CEUS of an 27-year old man with CTL in the effective group. Before treatment: **(A)** There was an enlarged left cervical lymph node with a maximum diameter of 3.5 cm and internal heterogeneity, and the echo of the surrounding soft tissue was not changed. **(B)** Elasticity score was 1. **(C)** CEUS showed irregular necrotic areas; After treatment: **(D)** The maximum diameter of the lymph node was 3.0 cm, and the echo of the surrounding soft tissue was hyperechoic. **(E)** Elasticity score was 2. **(F)** CEUS showed that the irregular necrotic area was reduced.

At 1–2 months after treatment, the maximum diameter of lymph nodes in the ineffective group was significantly larger than that in the effective group (*p* < 0.05). There were significant differences in pus changes, CDFI, elasticity scores, echo of surrounding tissues, changes in enlarged and non-enhanced areas after contrast enhancement between the two groups (Figures 1, 2) (*p* < 0.05). There was no significant difference in calcification and ring enhancement of lymph nodes between the two groups (*p* > 0.05), as shown in Table 4.

**Table 4:** Comparison of multimodality sonographic findings of the effective group and ineffective group after treatment.

	Effective (*n* = 46)	Ineffective (*n* = 15)	*p*
Maximum diameter (cm)	2.7 ± 0.7	4.1 ± 0.9	0.000
Pus			0.005
Absence	13	2	
Reduce	26	4	
Enlarge	7	9	
Strong echo			0.056
Absence	21	11	
Punctate	9	3	
Cluster-like	16	1	
CDFI			0.011
Hilar	2	0	
Peripheral	17	11	
Mixed	22	1	
Avascular	5	3	
Elastographyic score			0.001
1	2	7	
2	19	6	
3	18	2	
4	7	0	
Echo of surrounding soft-tissue			0.000
Enhanced	9	11	
Abscess formation	0	3	
Sinus	0	1	
Normal	37	0	
CEUS			
Enlargement	10	13	0.000
Rim-like enhancement	26	13	0.035
Non-enhancement area			0.000
Absence	6	0	
Reduce	31	1	
Enlarge	9	14	

## Discussion

Sputum smear and culture are important indicators for the early evaluation of pulmonary tuberculosis treatment response, which is crucial for the adjustment of treatment plan (9). However, the treatment response and evaluation indexes of TBL are different from those of pulmonary tuberculosis. At present, lymph node size is often used as the evaluation index, but studies have shown that changes in microcirculation in lymph nodes caused by tumor cells entering lymph nodes precede changes in lymph node size (10). In TBL, MTB enters the lymph nodes and causes an immune response, followed by the formation of tuberculous granulomas with or without caseous necrosis, which then liquefies and forms abscesses or sinuses. After effective treatment, MTB is suppressed, the lesions improve and calcification occurs (11). These different stages can be seen in ultrasound images (12). Therefore, we investigated the correlation between ultrasound images and the response to anti-TB chemotherapy of CTL.

Lymph node size is an influencing factor for predicting treatment response. The sensitivity and specificity of lymph node volume 44.15 cm^3^ in predicting non-response to anti-tuberculosis chemotherapy are 88.2 and 74.3%, respectively (13). However, Je BK et al. (14) showed that the initial size of lymph node tuberculosis was not correlated with drug treatment response and prognosis. This study showed no significant difference in the maximum diameter of lymph nodes between the two groups before treatment, and the difference between the two groups was statistically significant after treatment, so we agree with the latter view. Joo YH et al. found that the cutoff value of 0.36 for the necrotic zone ratio of lymph nodes had a sensitivity of 70.6% and a specificity of 71.4% for predicting the efficacy, which could be regarded as another factor related to the treatment response (13). In this study, there was no significant difference in the presence or absence of pus in the effective group before and after treatment, but the change in the range of pus between the ineffective group and the effective group was statistically significant. In the ineffective group, the range of pus was mostly increased, while in the effective group, the range of pus was mostly decreased. This suggests that an increase in pus may be indicative of treatment failure. The formation of pus is related to the progression of the disease, and the increase of pus during treatment indicates that the current drug regimen does not prevent the progression of the disease.

In this study, elastography showed that scores 1 and 2 were more common in the ineffective group, which may be related to the large amount of pus and soft texture in the lymph nodes. In the effective group, the pathological process gradually reversed to the proliferative or exudative phase due to the good response to drug treatment, and the pathological component was mainly solid tissue, so the scores of 2 and 3 were more common. However, there were many cases overlapping in the two scores between the two groups, and the value of elastic score in evaluating the response to treatment was limited. Strong echo in lymph nodes usually represents the transition of the lesion to the healing stage, and its size may increase with the disease course (15). This study showed no significant difference between the two groups, which may be related to the short treatment time of the included cases, and the correlation between this sign and prognosis could not be clearly determined at 1–2 months after treatment.

In the ultrasonographic image of CTL, enhanced surrounding soft tissue and enlargement after CEUS represent the inflammatory infiltration of the soft tissue around the lymph node (16). There were significant differences in these two signs between the two groups, and also in the effective group before and after treatment. The proportion of patients with post-contrast enlargement in the ineffective group (13/15, 86.7%) was significantly higher than that in the effective group (10/46, 21.7%). The inflammatory infiltration of CTL indicates the progressive stage of the disease. The enlargement of CTL during the treatment period after contrast-enhanced ultrasound may indicate that the lymph node responds poorly to the current drug treatment.

The non-enhanced area of CEUS in CTL includes pus and caseous necrosis, so it is mostly larger than pus, which can more accurately reflect the necrotic area in lymph nodes. The results of this study showed that the extent of necrosis in the ineffective group was more common than that before treatment, and the difference between the effective group and the ineffective group was statistically significant. Necrosis reflects the strong virulence of bacteria, weak immunity or severe allergic state, and its increase is another manifestation of poor response to drug treatment. The rim-like enhancement is an important sign in the diagnosis of CTL, which is due to the abnormal proliferation of granulation tissue and capillaries caused by the inflammatory reaction around the lymph nodes in the presence of various inflammatory chemokines (17). In this study, no significant difference was found in this sign before and after treatment in the effective group, but there was significant difference between the effective group and the ineffective group. We will continue to collect samples for further research on its predictive ability for anti-TB chemotherapy.

Notably, one of the cases showed enlargement of lymph nodes with abscess formation and increased echo of surrounding tissues in multimodal ultrasound examination at one month after treatment. The clinicians judged that there was a paradoxical response based on the comprehensive examination, and the original treatment was continued as maintenance therapy, and then it reached a stable state at six months. Studies have shown that about 15–25% of TB lymphadenitis patients with normal immune function have paradoxical reactions such as new or enlarged lymph nodes, cold abscess, systemic deterioration or focal tissue involvement during and even after treatment. The relevant mechanism remains unclear, and some scholars believe that it is an immune reaction to antigens released by dead TB bacilli (18, 19). Due to the small number of cases in this study, the image characteristics and identification of this type need to be further studied by increasing the sample size.

The main limitations of this study were the small sample size and insufficient analysis of special response of treatment, such as paradoxical responses. Meanwhile, this is an analysis of a single institution. However, the results are encouraging and provides a good basis for subsequent studies. we’ll accumulate cases and try to cooperate with other institutions to carry out further research.

In summary, multimodal ultrasound can provide in-depth information on the response of CTL to anti-TB chemotherapy. In addition to the size change of lymph node, the appearance of internal pus or non-enhancement area enlargement, enhanced echo of the surrounding tissue and enlargement after CEUS are related to poor prognosis, and maybe used to evaluate the response of anti-tuberculosis chemotherapy when the size change of lymph node is not obvious in individual treatment.

## Data availability statement

The raw data supporting the conclusions of this article will be made available by the authors, without undue reservation.

## Ethics statement

The studies involving human participants were reviewed and approved by Medical Ethics Committee of Affiliated Hangzhou Red Cross Hospital. The patients/participants provided their written informed consent to participate in this study. Written informed consent was obtained from the individual(s) for the publication of any potentially identifiable images or data included in this article.

## Author contributions

TY and YZ conceived and designed the project, analyzed the data, and drafted the initial manuscript. LZ, JX, JM, and XY collected and analysis the data and participated in patient management. All authors approved the final manuscript as submitted.

## Funding

This work was supported by the Medical Science and Technology Projects of Zhejiang Province (Nos. 2020KY732, 2023KY970).

## Conflict of interest

The authors declare that the research was conducted in the absence of any commercial or financial relationships that could be construed as a potential conflict of interest.

## Publisher’s note

All claims expressed in this article are solely those of the authors and do not necessarily represent those of their affiliated organizations, or those of the publisher, the editors and the reviewers. Any product that may be evaluated in this article, or claim that may be made by its manufacturer, is not guaranteed or endorsed by the publisher.
